# Supplementary material for: Single amino acid residue mediates reciprocal specificity in two mosquito odorant receptors
Source: eLife. 2022 Dec 13;11:e82922. doi: 10.7554/eLife.82922 (PMC9799979; doi:10.7554/eLife.82922)
Supplement: Supplementary file 1. [file elife-82922-supp1.docx]

**Supplementary file 1**

**Supplementary Tables 1 through 17**

**Supplementary file 1. Table 1.** Needleman-Wunsch percent identity and similarity of CquiOR10 to CquiOR2 transmembrane segments as defined by OCTOPUS.

|  | Percent Identity | Fraction identical residues | Percent Similarity | Fraction similar residues |
| --- | --- | --- | --- | --- |
| Full sequence | 49.5% | 139/382 | 71.7% | 274/382 |
| M1 | 45.5% | 10/22 | 68.2% | 15/22 |
| M2 | 52.4% | 11/21 | 81.0% | 17/21 |
| M3 | 66.7% | 16/24 | 79.2% | 19/24 |
| M4 | 71.9% | 23/32 | 78.1% | 25/32 |
| M5 | 61.3% | 19/31 | 80.6% | 25/31 |
| M6 | 47.6% | 10/21 | 85.7% | 18/21 |
| M7 | 81.0% | 17/21 | 85.7% | 18/21 |

**Supplementary file 1. Table 2.** Responses of CquiOR10Mx/CquiOrco-expressing oocytes to skatole and indole

| **Chimeric OR** | **The Most Potent Ligand** | **Sensitivity Relative to CquiOR10** |
| --- | --- | --- |
| WT | Skatole | **++** |
| M1,M2,M3,M4,M5,M6,M7 | No Response | **-** |
| M1,M2,M3,M4,M5,M6 | No Response | **-** |
| M1 | No Response | **-** |
| M2 | No Response | **-** |
| M3 | Skatole | **+** |
| M4 | Skatole | **+** |
| M5 | Skatole | **+++** |
| M6 | Skatole | **+++** |
| M7 | Skatole | **++** |
| M3,4,5,6,7 | Skatole | **++** |
| M3,4,5,6 | Skatole | **+** |
| M4,5,6 | Skatole | **+** |
| M3,5,6 | Skatole | **+** |
| M3,4,6 | Skatole | **+** |
| M3,4,5 | Skatole | **+** |
| M3,4 | Skatole | **++** |
| M3,5 | Skatole | **+++** |
| M3,6 | Skatole | **+** |
| M3,7 | Skatole | **++** |
| M4,5 | Skatole | **+++** |
| M4,6 | Skatole | **++** |
| M4,7 | Skatole | **++** |
| M5,6 | Skatole | **++** |
| M5,7 | Skatole | **+++** |
| M6,7 | Skatole | **+++** |
| M1,2 | No Response | **-** |
| M1,3 | No Response | **-** |
| M1,4 | No Response | **-** |
| M1,5 | No Response | **-** |
| M1,6 | No Response | **-** |
| M1,7 | No Response | **-** |
| M2,3 | No Response | **-** |
| M2,4 | No Response | **-** |
| M2,5 | No Response | **-** |
| M2,6 | No Response | **-** |
| M2,7 | Indole | **+** |

**Supplementary file 1. Table 3.** Lowest Rosetta interface score and lowest RMSD of 10 largest eugenol clusters docked to MhraOR5. ^†^

|  | Lowest interface score (REU) | RMSD (Å) | Cluster size |
| --- | --- | --- | --- |
| Cluster 1 | -17.8 | 0.75 | 1875 |
| Cluster 2 | -18.1 | 4.96 | 1283 |
| Cluster 3 | -19.2 | 5.05 | 912 |
| Cluster 4 | -17.6 | 2.79 | 860 |
| Cluster 5 | -18.3 | 2.82 | 665 |
| Cluster 6 | -17.9 | 2.93 | 656 |
| Cluster 7 | -17.8 | 2.79 | 563 |
| Cluster 8 | -18.3 | 0.55 | 510 |
| Cluster 9 | -21.2 | 2.37 | 322 |
| Cluster 10 | -17.9 | 2.82 | 305 |

^†^ Values should not be compared across columns since they originate from different predictions; values should only be compared within a column. REU: Rosetta Energy Units.

**Supplementary file 1. Table 4.** Lowest Rosetta interface score of 10 largest skatole clusters docked to CquiOR10 and CquiOR10-A73L

|  | CquiOR10 | | CquiOR10-A73L | |
| --- | --- | --- | --- | --- |
|  | Lowest interface score (REU) | Cluster size | Lowest interface score (REU) | Cluster size |
| Cluster 1 | -12.6 | 661 | -14.6 | 539 |
| Cluster 2 | -12.0 | 419 | -13.5 | 502 |
| Cluster 3 | -13.8 | 360 | -14.1 | 432 |
| Cluster 4 | -13.1 | 350 | -13.8 | 424 |
| Cluster 5 | -11.4 | 301 | -15.6 | 405 |
| Cluster 6 | -12.9 | 291 | -13.6 | 385 |
| Cluster 7 | -12.9 | 290 | -13.3 | 369 |
| Cluster 8 | -13.0 | 284 | -12.3 | 360 |
| Cluster 9 | -13.4 | 269 | -13.3 | 336 |
| Cluster 10 | -12.8 | 262 | -13.2 | 321 |

**Supplementary file 1. Table 5.** Lowest Rosetta interface score of 10 largest indole clusters docked to CquiOR10 and CquiOR10-A73L.

|  | CquiOR10 | | CquiOR10-A73L | |
| --- | --- | --- | --- | --- |
|  | Lowest interface score (REU) | Cluster size | Lowest interface score (REU) | Cluster size |
| Cluster 1 | -12.6 | 607 | -13.0 | 596 |
| Cluster 2 | -13.0 | 472 | -12.9 | 575 |
| Cluster 3 | -11.9 | 376 | -12.4 | 494 |
| Cluster 4 | -11.4 | 341 | -11.8 | 467 |
| Cluster 5 | -11.9 | 341 | -13.7 | 400 |
| Cluster 6 | -11.0 | 318 | -12.4 | 387 |
| Cluster 7 | -11.2 | 299 | -11.4 | 340 |
| Cluster 8 | -10.6 | 282 | -11.6 | 309 |
| Cluster 9 | -11.0 | 264 | -11.5 | 304 |
| Cluster 10 | -10.4 | 237 | -11.5 | 289 |

**Supplementary file 1. Table 6.** Hydrophobic interactions reported by the lowest interface-energy prediction from each cluster. The total PLIP-reported interactions^†^ and the total unique residues forming hydrophobic interactions with skatole/indole are reported.

| Cluster | CquiOR10 — skatole all PLIP-reported contacts | CquiOR10 — skatole unique residues | CquiOr10-A73L — skatole all PLIP-reported contacts | CquiOr10-A73L — skatole unique residues | CquiOR10 — indole all PLIP-reported contacts | CquiOR10 — indole unique residues | CquiOr10-A73L — indole all PLIP-reported contacts | CquiOr10-A73L — indole unique residues |
| --- | --- | --- | --- | --- | --- | --- | --- | --- |
| 1 | 8 | 5 | 5 | 5 | 4 | 4 | 4 | 4 |
| 2 | 6 | 5 | 6 | 6 | 4 | 4 | 5 | 4 |
| 3 | 8 | 7 | 4 | 4 | 5 | 5 | 5 | 5 |
| 4 | 6 | 5 | 6 | 6 | 5 | 5 | 3 | 3 |
| 5 | 6 | 4 | 5 | 5 | 3 | 2 | 5 | 4 |
| 6 | 5 | 3 | 5 | 5 | 6 | 5 | 5 | 5 |
| 7 | 3 | 3 | 7 | 5 | 6 | 5 | 2 | 2 |
| 8 | 8 | 6 | 4 | 4 | 3 | 2 | 3 | 3 |
| 9 | 7 | 6 | 5 | 5 | 6 | 3 | 5 | 5 |
| 10 | 6 | 5 | 5 | 4 | 4 | 3 | 2 | 2 |
| Median | 6.0 | 5.0 | 5.0 | 5.0 | 4.5 | 4.0 | 4.5 | 4.0 |
| Mean | 6.3 | 4.9 | 5.2 | 4.9 | 4.6 | 3.8 | 3.9 | 3.7 |
| Standard Deviation | 1.6 | 1.3 | 0.9 | 0.7 | 1.2 | 1.2 | 1.3 | 1.2 |

^†^ PLIP does not report the total number of hydrophobic interactions. Rather, the total hydrophobic interactions are clustered to 1) report interactions that are not π-stacking, 2) report the closest interaction distance a ligand atom interacts with binding site atoms, and 3) report the closest interaction distance a protein atom interacts with neighboring ligand atoms.

**Supplementary file 1. Table 7.** Lowest interface-energy predictions with Leu-73 hydrophobic interactions reported by PLIP.^†^

| Cluster | CquiOr10-A73L — skatole | CquiOr10-A73L — indole |
| --- | --- | --- |
| 2 | 1 | -- |
| 4 | 1 | -- |
| 5 | 1 | 1 |
| 6 | -- | 1 |
| 7 | 1 | -- |
| 9 | 1 | -- |

^†^ The lowest interface-energy prediction was used to represent each cluster.

**Supplementary file 1. Table 8.** Total PLIP-reported^†^ hydrophobic interactions* reported per-residue with indole/skatole.

| Residue | CquiOR10 — skatole | CquiOr10-A73L — skatole | CquiOR10 — indole | CquiOr10-A73L — indole | Mean skatole predictions | Mean indole predictions | Mean all structure predictions |
| --- | --- | --- | --- | --- | --- | --- | --- |
| Phe-66 | -- | -- | 1 | -- | -- | 1 | 1 |
| Ile-69 | 7 | 9 | 7 | 9 | 8 | 8 | 8 |
| Phe-70 | 1 | 2 | 1 | -- | 2 | 1 | 1 |
| Asn-72 | 7 | 5 | 5 | 4 | 6 | 4 | 5 |
| Ala/Leu73 | -- | 5 | -- | 2 | 5 | 2 | 4 |
| Ile-134 | 1 | 1 | -- | -- | 1 | -- | 1 |
| Tyr-138 | -- | 2 | 2 | -- | 2 | 2 | 2 |
| Tyr-152 | 1 | -- | -- | -- | 1 | -- | 1 |
| Tyr-185 | 10 | 10 | 8 | 10 | 10 | 9 | 10 |
| Ile-186 | -- | -- | 2 | -- | -- | 2 | 2 |
| Phe-261 | 6 | 5 | 6 | 5 | 6 | 6 | 6 |
| Tyr-287 | 8 | 6 | 2 | 3 | 7 | 2 | 5 |
| Ile-291 | 4 | 4 | 1 | 2 | 4 | 2 | 3 |
| Gln-294 | 3 | -- | 3 | 2 | 3 | 2 | 3 |

^†^ PLIP does not report the total number of hydrophobic interactions. Rather, the total hydrophobic interactions are clustered to 1) report interactions that are not π-stacking, 2) report the closest interaction distance a ligand atom interacts with binding site atoms, and 3) report the closest interaction distance a protein atom interacts with neighboring ligand atoms.

* Values represent the total number of representative predictions interacting with the specified residue. Each cluster is represented by the lowest interface-energy prediction.

**Supplementary file 1. Table 9.** Hydrogen bond interactions reported by the lowest interface-energy prediction from each cluster. The total PLIP-reported interactions^†^ and the total unique residues forming hydrogen bonds with skatole/indole are reported.

| Cluster | CquiOR10 — skatole all PLIP-reported contacts | CquiOR10 — skatole unique residues | CquiOr10-A73L — skatole all PLIP-reported contacts | CquiOr10-A73L — skatole unique residues | CquiOR10 — indole all PLIP-reported contacts | CquiOR10 — indole unique residues | CquiOr10-A73L — indole all PLIP-reported contacts | CquiOr10-A73L — indole unique residues |
| --- | --- | --- | --- | --- | --- | --- | --- | --- |
| 1 | -- | -- | -- | -- | -- | -- | 1 | 1 |
| 2 | -- | -- | -- | -- | -- | -- | -- | -- |
| 3 | -- | -- | -- | -- | 1 | 1 | -- | -- |
| 4 | -- | -- | 1 | 1 | -- | -- | -- | -- |
| 5 | -- | -- | 1 | 1 | -- | -- | 1 | 1 |
| 6 | -- | -- | -- | -- | -- | -- | -- | -- |
| 7 | 2 | 1 | -- | -- | 2 | 1 | -- | -- |
| 8 | 2 | 1 | -- | -- | 1 | 1 | -- | -- |
| 9 | -- | -- | -- | -- | 1 | 1 | -- | -- |
| 10 | -- | -- | -- | -- | -- | -- | -- | -- |
| Median | 0.0 | 0.0 | 0.0 | 0.0 | 0.0 | 0.0 | 0.0 | 0.0 |
| Mean | 0.4 | 0.2 | 0.2 | 0.2 | 0.5 | 0.4 | 0.2 | 0.2 |
| Standard Deviation | 0.8 | 0.4 | 0.4 | 0.4 | 0.7 | 0.5 | 0.4 | 0.4 |

^†^ Hydrogen bond interactions reported by PLIP were filtered using previously reported bond distances (Bissantz et al., 2010).

**Supplementary file 1. Table 10.** Total PLIP-reported^†^ hydrogen bond interactions reported per-residue with indole/skatole.

| Residue | CquiOR10 — skatole | CquiOr10-A73L — skatole | CquiOR10 — indole | CquiOr10-A73L — indole | Mean skatole predictions | Mean indole predictions | Mean all structure predictions |
| --- | --- | --- | --- | --- | --- | --- | --- |
| Ile-69 | -- | -- | 1 | -- | -- | 1 | 1 |
| Asn-72 | -- | 1 | -- | -- | 1 | -- | 1 |
| Tyr-138 | -- | -- | 1 | -- | -- | 1 | 1 |
| Tyr-287 | -- | -- | 1 | -- | -- | 1 | 1 |
| Gln-294 | 2 | 1 | 1 | 2 | 2 | 2 | 2 |

^†^ Hydrogen bond interactions reported by PLIP were filtered using previously reported bond distances(Bissantz et al., 2010).

**Supplementary file 1. Table 11.** Descriptions of each hydrogen bond reported by each cluster’s representative^†^ prediction.

| Structure — Ligand | Cluster | Residue | Donor* | Acceptor* | Donor-Acceptor Distance (Å) |
| --- | --- | --- | --- | --- | --- |
| CquiOR10 — skatole | 7 | Gln-294 | Gln-294 sidechain, Nam | Skatole, Npl | 3.1 |
| CquiOR10 — skatole | 7 | Gln-294 | Skatole, Npl | Gln-294 sidechain, O2 | 2.9 |
| CquiOR10 — skatole | 8 | Gln-294 | Gln-294 sidechain, Nam | Skatole, Npl | 3.1 |
| CquiOR10 — skatole | 8 | Gln-294 | Skatole, Npl | Gln-294 sidechain, O2 | 2.7 |
| CquiOR10-A73L — skatole | 4 | Asn-72 | Asn-72 sidechain, Nam | Skatole, Npl | 3.2 |
| CquiOR10-A73L — skatole | 5 | Gln-294 | Skatole, Npl | Gln-294 sidechain, O2 | 2.8 |
| CquiOR10 — indole | 3 | Tyr-287 | Tyr-287 sidechain, O3 | Indole, Npl | 3.0 |
| CquiOR10 — indole | 7 | Gln-294 | Gln-294 sidechain, Nam | Indole, Npl | 3.1 |
| CquiOR10 — indole | 7 | Gln-294 | Indole, Npl | Gln-294 sidechain, O2 | 2.8 |
| CquiOR10 — indole | 8 | Ile-69 | Indole, Npl | Ile-69 backbone, O2 | 3.1 |
| CquiOR10 — indole | 9 | Tyr-138 | Indole, Npl | Tyr-138 sidechain, O3 | 2.8 |
| CquiOR10-A73L — indole | 1 | Gln-294 | Indole, Npl | Gln-294 sidechain, O2 | 2.9 |
| CquiOR10-A73L — indole | 5 | Gln-294 | Indole, Npl | Gln-294 sidechain, O2 | 3.0 |

^†^ The lowest interface-energy prediction was used to represent each cluster.

* The atom type definition is derived from IDATM nomenclature (Meng & Lewis, 1991).

**Supplementary file 1. Table 12.** Parallel pi-stacking interactions reported by the lowest interface-energy prediction from each cluster. The total PLIP-reported interactions^†^ and the total unique residues forming parallel pi-stacking interactions with skatole/indole are reported.

| Cluster | CquiOR10 — skatole all PLIP-reported contacts | CquiOR10 — skatole unique residues | CquiOr10-A73L — skatole all PLIP-reported contacts | CquiOr10-A73L — skatole unique residues | CquiOR10 — indole all PLIP-reported contacts | CquiOR10 — indole unique residues | CquiOr10-A73L — indole all PLIP-reported contacts | CquiOr10-A73L — indole unique residues |
| --- | --- | --- | --- | --- | --- | --- | --- | --- |
| 1 | -- | -- | 1 | 1 | -- | -- | 1 | 1 |
| 2 | -- | -- | -- | -- | -- | -- | 1 | 1 |
| 3 | -- | -- | -- | -- | -- | -- | 1 | 1 |
| 4 | -- | -- | 1 | 1 | -- | -- | -- | -- |
| 5 | -- | -- | -- | -- | 2 | 2 | 1 | 1 |
| 6 | -- | -- | -- | -- | -- | -- | -- | -- |
| 7 | -- | -- | -- | -- | -- | -- | -- | -- |
| 8 | -- | -- | -- | -- | -- | -- | 1 | 1 |
| 9 | -- | -- | -- | -- | -- | -- | 1 | 1 |
| 10 | -- | -- | -- | -- | -- | -- | 2 | 1 |
| Median | 0.0 | 0.0 | 0.0 | 0.0 | 0.0 | 0.0 | 1.0 | 1.0 |
| Mean | 0.0 | 0.0 | 0.2 | 0.2 | 0.2 | 0.2 | 0.8 | 0.7 |
| Standard Deviation | 0.0 | 0.0 | 0.4 | 0.4 | 0.6 | 0.6 | 0.6 | 0.5 |

^†^ Parallel pi-stacking interactions were filtered by previously reported bond distances (Bissantz et al., 2010). No perpendicular pi-stacking interactions met filtering distance criteria.

**Supplementary file 1. Table 13.** Total PLIP-reported parallel pi-stacking interactions^†^ reported per-residue with indole/skatole.

| Residue | CquiOR10 — skatole | CquiOr10-A73L — skatole | CquiOR10 — indole | CquiOr10-A73L — indole | Mean skatole predictions | Mean indole predictions | Mean all structure predictions |
| --- | --- | --- | --- | --- | --- | --- | --- |
| Tyr-138 | -- | -- | 1 | -- | -- | 1 | 1 |
| Tyr-152 | -- | -- | 1 | -- | -- | 1 | 1 |
| Tyr-185 | -- | 2 | -- | 8 | 2 | 8 | 5 |

^†^ Parallel pi-stacking interactions were filtered by previously reported bond distances(Bissantz et al., 2010). No perpendicular pi-stacking interactions met filtering distance criteria.

**Supplementary file 1. Table 14.** Descriptions of each parallel pi-stacking interaction reported by each cluster’s representative^†^ prediction.

| Structure — Ligand | Cluster | Residue | Central ring distance (Å)* |
| --- | --- | --- | --- |
| CquiOR10-A73L — skatole | 1 | Tyr-185 | 3.6 |
| CquiOR10-A73L — skatole | 4 | Tyr-185 | 3.4 |
| CquiOR10 — indole | 5 | Tyr-138 | 3.5 |
| CquiOR10 — indole | 5 | Tyr-152 | 3.4 |
| CquiOR10-A73L — indole | 1 | Tyr-185 | 3.4 |
| CquiOR10-A73L — indole | 2 | Tyr-185 | 3.6 |
| CquiOR10-A73L — indole | 3 | Tyr-185 | 3.6 |
| CquiOR10-A73L — indole | 5 | Tyr-185 | 3.4 |
| CquiOR10-A73L — indole | 8 | Tyr-185 | 3.6 |
| CquiOR10-A73L — indole | 9 | Tyr-185 | 3.6 |
| CquiOR10-A73L — indole | 10 | Tyr-185 | 3.6 |
| CquiOR10-A73L — indole | 10 | Tyr-185 | 3.6 |

^†^ The lowest interface-energy prediction was used to represent each cluster.

* The central distance is computed by two centroids positioned at the center each aromatic ring.

**Supplementary file 1. Table 15.** Cartesian coordinates of C-alpha atoms at position 73 from CquiOR10/A73L docking. The lowest interface-scoring model was used from each cluster. Models were aligned to helix S7b prior to recording position.

|  | Skatole | | | | | | Indole | | | | | |
| --- | --- | --- | --- | --- | --- | --- | --- | --- | --- | --- | --- | --- |
|  | CquiOR10 | | | CquiOr10-A73L | | | CquiOR10 | | | CquiOr10-A73L | | |
|  | x | y | z | x | y | z | x | y | z | x | y | z |
| Cluster 1 | 164.917 | 184.462 | 155.034 | 165.788 | 183.953 | 155.051 | 164.914 | 184.490 | 155.036 | 165.797 | 183.967 | 155.054 |
| Cluster 2 | 164.936 | 184.513 | 155.026 | 165.801 | 183.981 | 155.051 | 164.928 | 184.470 | 155.066 | 165.808 | 183.975 | 155.054 |
| Cluster 3 | 164.934 | 184.465 | 155.063 | 165.795 | 183.961 | 155.057 | 164.967 | 184.470 | 155.012 | 165.800 | 183.964 | 155.058 |
| Cluster 4 | 164.925 | 184.485 | 155.038 | 165.807 | 183.970 | 155.061 | 164.920 | 184.464 | 155.052 | 165.796 | 183.976 | 155.055 |
| Cluster 5 | 164.934 | 184.436 | 155.038 | 165.885 | 184.086 | 155.111 | 164.938 | 184.488 | 155.016 | 165.872 | 184.010 | 155.119 |
| Cluster 6 | 164.914 | 184.533 | 155.032 | 165.790 | 184.023 | 155.039 | 164.917 | 184.541 | 155.034 | 165.803 | 183.971 | 155.048 |
| Cluster 7 | 164.953 | 184.446 | 155.039 | 165.795 | 183.972 | 155.046 | 164.940 | 184.472 | 155.049 | 165.791 | 183.968 | 155.060 |
| Cluster 8 | 164.927 | 184.477 | 155.043 | 165.776 | 183.949 | 155.063 | 164.937 | 184.489 | 155.034 | 165.870 | 184.015 | 155.113 |
| Cluster 9 | 164.923 | 184.487 | 155.036 | 165.915 | 184.044 | 155.142 | 164.935 | 184.534 | 155.035 | 165.792 | 183.957 | 155.051 |
| Cluster 10 | 164.931 | 184.439 | 155.050 | 165.797 | 184.009 | 155.058 | 164.932 | 184.442 | 155.045 | 165.790 | 183.956 | 155.052 |
| Mean | 164.929 | 184.474 | 155.040 | 165.815 | 183.995 | 155.068 | 164.933 | 184.486 | 155.038 | 165.812 | 183.976 | 155.066 |
| Standard Deviation (Å) | 0.011 | 0.032 | 0.010 | 0.0461 | 0.045 | 0.032 | 0.015 | 0.031 | 0.016 | 0.032 | 0.020 | 0.026 |

**Supplementary file 1. Table 16.** Mean distances (Å) of C-alpha atoms at position 73 from CquiOR10/A73L docking using skatole and indole. The lowest interface-scoring model was used from each cluster. Models were aligned to helix S7b prior to distance calculation.

| Protein — Ligand | CquiOR10 — skatole | CquiOr10-A73L — skatole | CquiOR10 — indole | CquiOr10-A73L — indole |
| --- | --- | --- | --- | --- |
| CquiOR10 — skatole | -- | -- | -- | -- |
| CquiOr10-A73L — skatole | 1.007 | -- | -- | -- |
| CquiOR10 — indole | 0.012 | 1.010 | -- | -- |
| CquiOr10-A73L — indole | 1.014 | 0.019 | 1.017 | -- |

**Supplementary file 1. Table 17.** CquiOR2 residues corresponding to CquiOR10 from Clustal Omega multiple sequence alignment and Needleman-Wunsch pairwise alignment.

| CquiOR10  Residue | CquiOR2  ClustalO | CquiOR2  Needleman-Wunsch |
| --- | --- | --- |
| Phe-66 | Phe-67 | Phe-67 |
| Ile-69 | Leu-70 | Leu-70 |
| Phe-70 | Tyr-71 | Tyr-71 |
| Asn-72 | Asn-73 | Asn-73 |
| Ala/Leu73 | Leu-74 | Leu-74 |
| Ile-134 | Ile-133 | Ile-133 |
| Tyr-138 | Phe-137 | Phe-137 |
| Tyr-152 | Tyr-151 | Tyr-151 |
| Tyr-185 | Tyr-184 | Tyr-184 |
| Ile-186 | Ile-185 | Ile-185 |
| Phe-261 | Phe-258 | Phe-258 |
| Tyr-287 | Tyr-284 | Tyr-284 |
| Ile-291 | Ile-288 | Ile-288 |
| Gln-294 | Gln-291 | Gln-291 |
