# Supplementary material for: Single amino acid residue mediates reciprocal specificity in two mosquito odorant receptors
Source: eLife. 2022 Dec 13;11:e82922. doi: 10.7554/eLife.82922 (PMC9799979; doi:10.7554/eLife.82922)
Supplement: Supplementary file 2. [file elife-82922-supp2.docx]

**Supplementary file 2**

**Supplementary file 2. Protocol Capture**

**S 1. Generating conformer library with OpenEye Omega and RosettaLigand params – Bash executable**

| 1  2  ∙  3  4  5  6  7  8  ∙  9  10  11  12  13  14  15  16  17  18  19  20  21  22  23  ∙  24  ∙  25  ∙  26  27  28  29  30  31  32  33  ∙  34  35  36  37  38  39  40  41  42 | #!/bin/bash  # New to OpenEye? Install the openeye toolkit with Conda.  (Read the OpenEye toolkit README)  # Using Python2.7? Look for pip install packages at: https://anaconda.org/OpenEye  rosetta_src={YOUR_FOLDER_PATH}/rosetta_bin_linux_2021.07.61567_bundle/main/source  openeye_bin={YOUR_FOLDER_PATH}/openeye/bin  **if** [ $# -lt 1 ]; **then**  echo "USAGE: generate-ligand-conformers.sh  <ligand name only (input in .mol2 format)> "  exit  **fi**  drug=$1  dir=**$(**echo **${**PWD**})**  set -v  # Set up directory structure  mkdir -p ligand  # Make ligands  pushd ligand  mkdir -p {fa,cen}/{conf1,confs,kins,withxtal}  omega="${openeye_bin}/omega2 -includeInput -commentEnergy"  $omega -in $dir/$drug.mol2 -out $drug.omega.mol2 -prefix _$drug  python **${**rosetta_src**}**/src/apps/public/ligand_docking/assign_charges.py  < $drug.omega.mol2 > $drug.am1bcc.mol2  python **${**rosetta_src**}**/scripts/python/public/molfile_to_params.py -c -nX00  -p$drug -k$drug.kin $drug.am1bcc.mol2  cat **${**drug**}**_????.fa.pdb \| gzip -c > fa/withxtal/**${**drug**}**_confs.fa.pdb.gz &&  ( [ -f **${**drug**}**_0002.fa.pdb ] \|\| cp **${**drug**}**_0001.fa.pdb **${**drug**}**_0002.fa.pdb )  mv **${**drug**}**_0001.fa.pdb fa/conf1/  cat **${**drug**}**_????.fa.pdb \| gzip -c > fa/**${**drug**}**_confs.fa.pdb.gz  mv **${**drug**}**_????.fa.pdb fa/confs/  echo "PDB_ROTAMERS ${drug}_confs.fa.pdb" >> $drug.fa.params  cp $drug.fa.params fa/withxtal/  mv $drug.fa.params fa/  mv $drug.fa.kin fa/kins/  cat **${**drug**}**_????.cen.pdb \| gzip -c > cen/withxtal/**${**drug**}**_confs.cen.pdb.gz &&  ( [ -f **${**drug**}**_0002.cen.pdb ] \|\| cp **${**drug**}**_0001.cen.pdb **${**drug**}**_0002.cen.pdb )  mv **${**drug**}**_0001.cen.pdb cen/conf1/  cat **${**drug**}**_????.cen.pdb \| gzip -c > cen/**${**drug**}**_confs.cen.pdb.gz  mv **${**drug**}**_????.cen.pdb cen/confs/  echo 'PDB_ROTAMERS $drug_confs.cen.pdb' >> $drug.cen.params  cp $drug.cen.params cen/withxtal/  mv $drug.cen.params cen/  mv $drug.cen.kin cen/kins/  popd |
| --- | --- |

**S2. RosettaLigand – Bash executable**

| 1  2  3  4  5  6  7  8  9  10  11  12  13  14  15  16  ∙  17 | #!/bin/bash  ${rosetta_bin}/rosetta_scripts.static.linuxgccrelease **\**  -in:path:database $rosetta_database **\**  -s ./seed_files/**${**protein_ligand_complex.pdb**}** **\**  -parser:protocol ./seed_files/dock.xml **\**  -extra_res_fa ./seed_files/**${**file_handle**}**.params **\**  -ex1 **\**  -ex2 **\**  -no_optH false **\**  -flip_HNQ true **\**  -ignore_ligand_chi true **\**  -nstruct 10 **\**  -overwrite **\**  -out:pdb true **\**  -out:prefix **${**array_prefix**}**_ **\**  -out:file:scorefile **${**array_prefix**}**_Rosetta_ligand_**${**file_handle**}**.sc |
| --- | --- |

**S3. RosettaLigand Docking – XML script**

| 1  2  3  4  5  6  7  8  9  10  ∙  11  ∙  12  ∙  13  14  15  16  ∙  17  ∙  18  ∙  19  20  21  22  ∙  23  ∙  ∙  24  25  26  27  28  29  30  31  ∙  32  ∙  33  ∙  34  ∙  35  36  37  38  39  40  41  42  43  44 | <ROSETTASCRIPTS>  <SCOREFXNS>  <ScoreFunction name="ligand_soft_rep" weights="ligand_soft_rep">  </ScoreFunction>  <ScoreFunction name="hard_rep" weights="ligand">  </ScoreFunction>  </SCOREFXNS>  <LIGAND_AREAS>  <LigandArea name="inhibitor_dock_sc" chain="X" cutoff="6.0"  add_nbr_radius="true" all_atom_mode="false"/>  <LigandArea name="inhibitor_final_sc" chain="X" cutoff="6.0"  add_nbr_radius="true" all_atom_mode="false"/>  <LigandArea name="inhibitor_final_bb" chain="X" cutoff="7.0"  add_nbr_radius="false" all_atom_mode="true" Calpha_restraints="0.3"/>  </LIGAND_AREAS>  <INTERFACE_BUILDERS>  <InterfaceBuilder name="side_chain_for_docking"  ligand_areas="inhibitor_dock_sc"/>  <InterfaceBuilder name="side_chain_for_final"  ligand_areas="inhibitor_final_sc"/>  <InterfaceBuilder name="backbone"  ligand_areas="inhibitor_final_bb" extension_window="3"/>  </INTERFACE_BUILDERS>  <MOVEMAP_BUILDERS>  <MoveMapBuilder name="docking"  sc_interface="side_chain_for_docking" minimize_water="false"/>  <MoveMapBuilder name="final"  sc_interface="side_chain_for_final" bb_interface="backbone"  minimize_water="false"/>  </MOVEMAP_BUILDERS>  <SCORINGGRIDS ligand_chain="X" width="15">  <ClassicGrid grid_name="classic" weight="1.0"/>  </SCORINGGRIDS>  <MOVERS>  <Transform name="transform" chain="X" box_size="7.0"  move_distance="0.2" angle="20" cycles="500" repeats="1" temperature="5"/>  <HighResDocker name="high_res_docker" cycles="6"  repack_every_Nth="3" scorefxn="ligand_soft_rep" movemap_builder="docking"/>  <FinalMinimizer name="final" scorefxn="hard_rep"  movemap_builder="final"/>  <InterfaceScoreCalculator name="add_scores" chains="X"  scorefxn="hard_rep"/>  </MOVERS>  <PROTOCOLS>  <Add mover_name="transform"/>  <Add mover_name="high_res_docker"/>  <Add mover_name="final"/>  <Add mover_name="add_scores"/>  </PROTOCOLS>  </ROSETTASCRIPTS> |
| --- | --- |

**S4. Example hdbscan clustering – Bash executable**

| 1  2  3  4  ∙  5 | #!/bin/bash  Biomol2Clust=/home/user/Software/Biomol2Clust_v.1.3/main.py  python3 $Biomol2Clust method=hdbscan noh=true input=./cluster_inputs  min_cluster_size=50 output=./hdbscan_50 |
| --- | --- |

**S5. Example PLIP analysis – Bash executable**

| 1  2  3  4  5  6  7 | #!/bin/bash  plip=/home/user/anaconda2/envs/plip/bin/plip  pdb={YOUR PDB INPUT}  plip -yvpt –nohydro -f $pdb |
| --- | --- |
